# The impact of pelvic floor electrical stimulation on vaginal microbiota and immunity

**DOI:** 10.3389/fcimb.2022.1006576

**Published:** 2022-09-27

**Authors:** Yakun Zhang, He Yang, Chi Zhang, Li Lin, Wenlan Yang, Guangwu Xiong, Guolan Gao

**Affiliations:** ^1^ Savaid Medical School, University of Chinese Academy of Sciences, Beijing, China; ^2^ Department of Obstetrics and Gynecology, Peking University International Hospital, Beijing, China

**Keywords:** vaginal microbiota, electrical stimulation, 16S rRNA, pelvic floor muscle, local cytokine

## Abstract

Pelvic floor electrical stimulation (ES) is an effective treatment for pelvic floor dysfunction. However, the impact of ES on vaginal microbiota and local inflammatory response is yet poorly understood. Therefore, we designed a longitudinal study to investigate the impact of ES on vaginal microbiota and cytokines. A total of 170 participants were recruited into the study at Peking University International Hospital, Beijing, China, from December 2021 to April 2022. They were divided into two groups concerning the follow-up: long-term cohort (*n* = 147) following up to seven treatment sessions and short-term cohort (*n* = 23) following up to 7 h after a 30-min treatment. Paired vaginal discharge samples were collected from 134 individuals. Vaginal microbiota was characterized by 16S rRNA sequencing, and local cytokines concentrations were detected by the cytometric bead array method. A significant increase in the relative abundance of *Lactobacillus* spp. was observed after ES treatment (*P* < 0.001). In addition, *L. crispatus* (*P* = 0.012) and *L. gasseri* (*P* = 0.011) also increased significantly. Reduced microbial diversity was observed in the vaginal microbiota after the treatment. In the long-term cohort, a significant downregulation of IFN-γ, IL-2, IL-4, IL-10, IL-17A, and TNF-α was compared with baseline. However, the short-term cohort presented with an elevated IL-6 level at 7 h after the treatment. In conclusion, this study suggested that transvaginal electrical stimulation might help to restore and maintain a healthy vaginal microbiota dominated by *Lactobacillus*, reducing the risk of vaginal inflammation.

## Introduction

Pelvic floor electrical stimulation (ES) is an effective method to increase voluntary pelvic muscle contractions and enhance muscle strength, and even muscles suffering from severe impairment can benefit to regain the ability to perform voluntary contractions (Li et al., 2020). Transvaginal electrical stimulation (TVES) is a passive treatment that can excite nerves and stimulate pelvic floor muscles by sending mild electrical currents and has proven efficacy in the treatment of pelvic floor dysfunction (PFD) like stress urinary incontinence, fecal incontinence, pelvic organ prolapse (POP), and sexual dysfunction (Yang et al., 2017; Silantyeva et al., 2021).

The vaginal microbiome of healthy women is characterized by low microbial diversity and *Lactobacillus* predominance (Ravel et al., 2011). *Lactobacillus* adhere to the vaginal epithelial surface and play a protective role in preventing infection (Chee et al., 2020). An abnormal microbiome has been associated with increased risk of reproductive tract inflammation, abortion, and human papillomavirus (HPV) infection (Chen et al., 2020). Therefore, a healthy and balanced vaginal microbiota is crucial for reproductive health. However, the effect of TVES on vaginal microbiome has not been reported.

The effect of ES on various microorganisms such as *Escherichia coli*, *Staphylococcus aureus*, and *Pseudomonas aeruginosa* can be inhibitory, stimulatory, or none depending on the parameters and modes selected (Caubet et al., 2004; Dusane et al., 2019; Lee et al., 2021). Studies showed that the mechanisms of ES for promoting wound healing might include increased angiogenesis and circulation as well as a direct antibacterial effect of ES (Gentzkow, 1993; Asadi and Torkaman, 2014; Ashrafi et al., 2017). Wang *et al*. found that electroacupuncture treatment not only helped alleviate knee pain from knee osteoarthritis but also influenced the gut microbiota by reducing the abundance of pathogenic bacteria, such as *Streptococcus*, and increasing the abundance of beneficial bacteria, including *Agathobacter* and *Bacteroide*, in the gut (Wang et al., 2021).

Cytokine concentrations may reflect, to some degree, conditions on local inflammatory and pathological conditions. Increased inflammatory cytokines in the cervix and vagina were associated with an increased risk of HIV-1 transmission (Mitchell and Marrazzo, 2014). A recent study showed that HPV-infected patients reported elevated levels of cytokines like IL-1α (interleukin-1α), IL-1β, IL-6, IL-10, IL-8, IL-17A, INF-γ (interferon-γ), and TNF-α (tumor necrosis factor). The increased level of those cytokines was associated with the severity of disease from low-grade squamous intraepithelial lesion and high-grade squamous intraepithelial lesion to cancer (Otani et al., 2019). Inflammation leads to disruption of the vaginal epithelial barrier and reduction in antimicrobial peptide secretion, which is linked to increased susceptibility to sexually transmitted infections (STIs) (Gopinath and Iwasaki, 2015).

A study showed that moderate low-frequency transcutaneous neuromuscular ES slightly strengthened the quadriceps femoris muscle while producing no changes in the measured blood immunological parameters (Kopitar et al., 2012). Another study found the muscle strength of patients to have improved after high- or low-frequency peripheral neuromuscular ES, while the inflammatory processes seemed more linked to the ES strategies adopted (Bruggemann et al., 2017). However, no evidence of whether ES may induce local inflammatory responses in vaginal epithelium has been found.

In summary, few studies have actually reported the impact of ES on vaginal microbiota and the immune microenvironment. It remains to be explored whether TVES will affect the dominance of *Lactobacillus* and inflammatory regulation. Therefore, we designed a longitudinal controlled cohort study to investigate the effects of pelvic floor ES on vaginal micro-ecology.

## Results

### Participants’ characteristics

**Figure 1:**
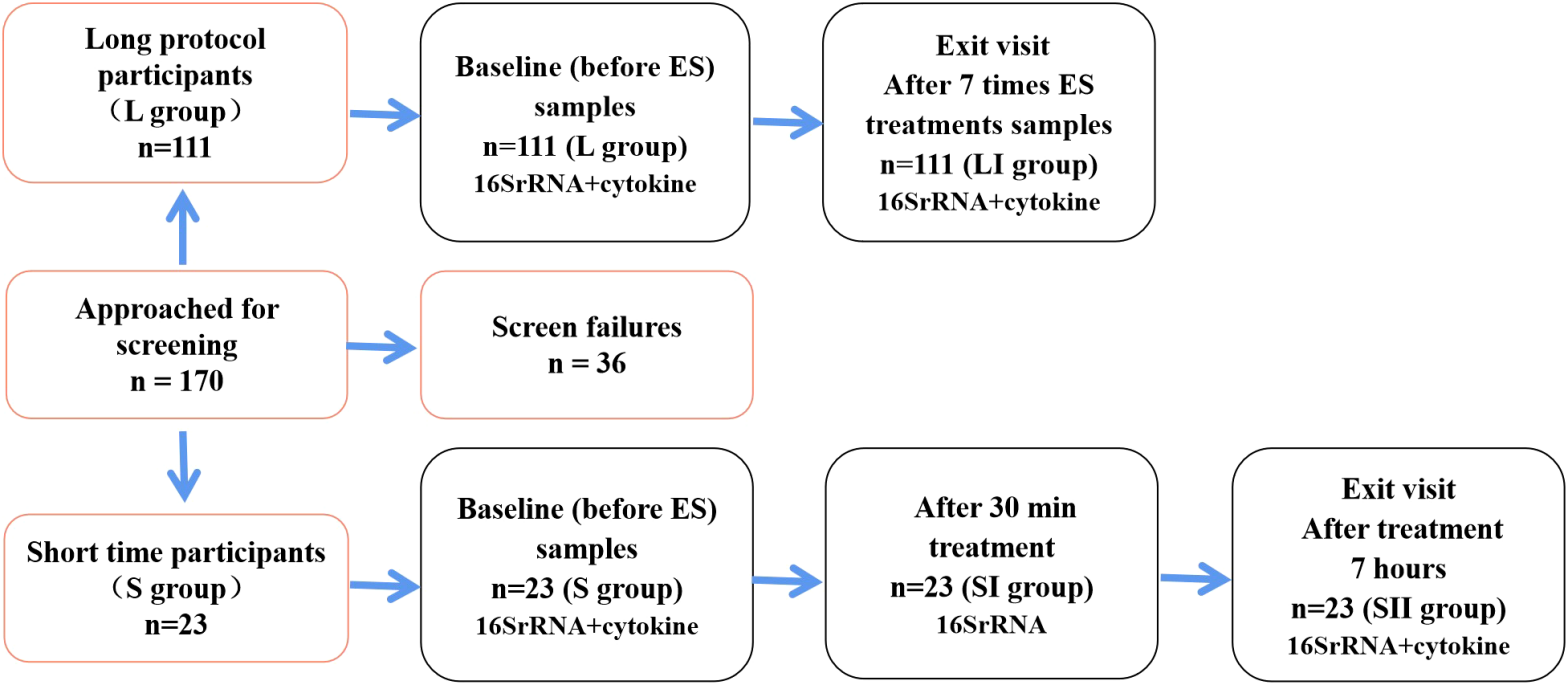
Flow diagram showing the distribution of participants using different follow-up protocols and the number of samples that completed each study visit, respectively.

The participants were recruited from the Department of Obstetrics and Gynecology of Peking University International Hospital from December 2021 to April 2022. A total of 170 individuals were recruited into the longitudinal study, while 36 women failed to meet the inclusion criteria and were excluded. Furthermore, 134 women were enrolled and completed their follow-up with paired vaginal secretion samples tested (**Figure 1**). Of these enrolled patients, 120 had been diagnosed with urinary incontinence, weakened pelvic floor muscles after childbirth, pelvic pain, vaginal laxity, or POP, and they were randomly divided into two groups, namely, the long-term group (L group, *n* = 111) (**Supplementary Table S1**) and the short-term cohort group (S group, *n* = 9). The rest of the 14 healthy subjects were classified into the S group receiving 30 min of ES treatment. The 23 women in the S group completed a 7-h follow-up (**Supplementary Table S2**).

After seven treatment sessions, the rate of effectiveness of the L group was 81.1% as determined by the improvement of subjective symptoms or pelvic floor muscle functions (**Table 1**).

**Table 1:** Characteristics of all participants (*n* = 134).

Characteristic	L group (*n* = 111)	S group (*n* = 23)
Age, mean (SD), years	33.4 (5.07)	31.5 (4.26)
Sexual intercourse during treatments, *n* (%)	44 (39.6%)	0
Sexual partners during treatments ≥2, *n* (%)	0	–
Condom use during treatments, *n* (%)	42 (95.4%)	–
Menstruation during treatments, *n* (%)	41 (36.9%)	0
Mean current intensity, median (IQR), mA	38 (32–50)	29 (28–31)
Improvement of symptoms or muscle strength after treatments, *n* (%)	90 (81.1%)	–

### Vaginal microbiota composition

All of the 291 samples utilizing 16S rRNA sequencing passed the quality control measures with a median sequencing read depth of 85,433 reads [interquartile range (IQR) 81,720–89,094]. L group and S group acquired 34,777 and 16,437 operational taxonomic units (OTUs), respectively. The detailed relative abundances of all samples are shown in **Supplementary Table S3**.

Of the *Lactobacillus* spp. in this study, *Lactobacillus iners*, *Lactobacillus crispatus*, and *Lactobacillus gasseri* were the most prevalent species in the vagina. The vaginal microbiota in the long-term cohort had lower relative abundance of *Lactobacillus* and higher diversity in flora composition (compared by Chao1 and Shannon index, *P* < 0.01) compared with those of the short-term cohort (37.58 *vs*. 63.49%), which was associated with PFD, or the postpartum recovery phases that the L group participants were in. PCoA based on Bray–Curtis was used to display the composition differences between samples (**Supplementary Figure S1**).

### Impact of seven sessions of ES treatment on vaginal microbiota

**Figure 2:**
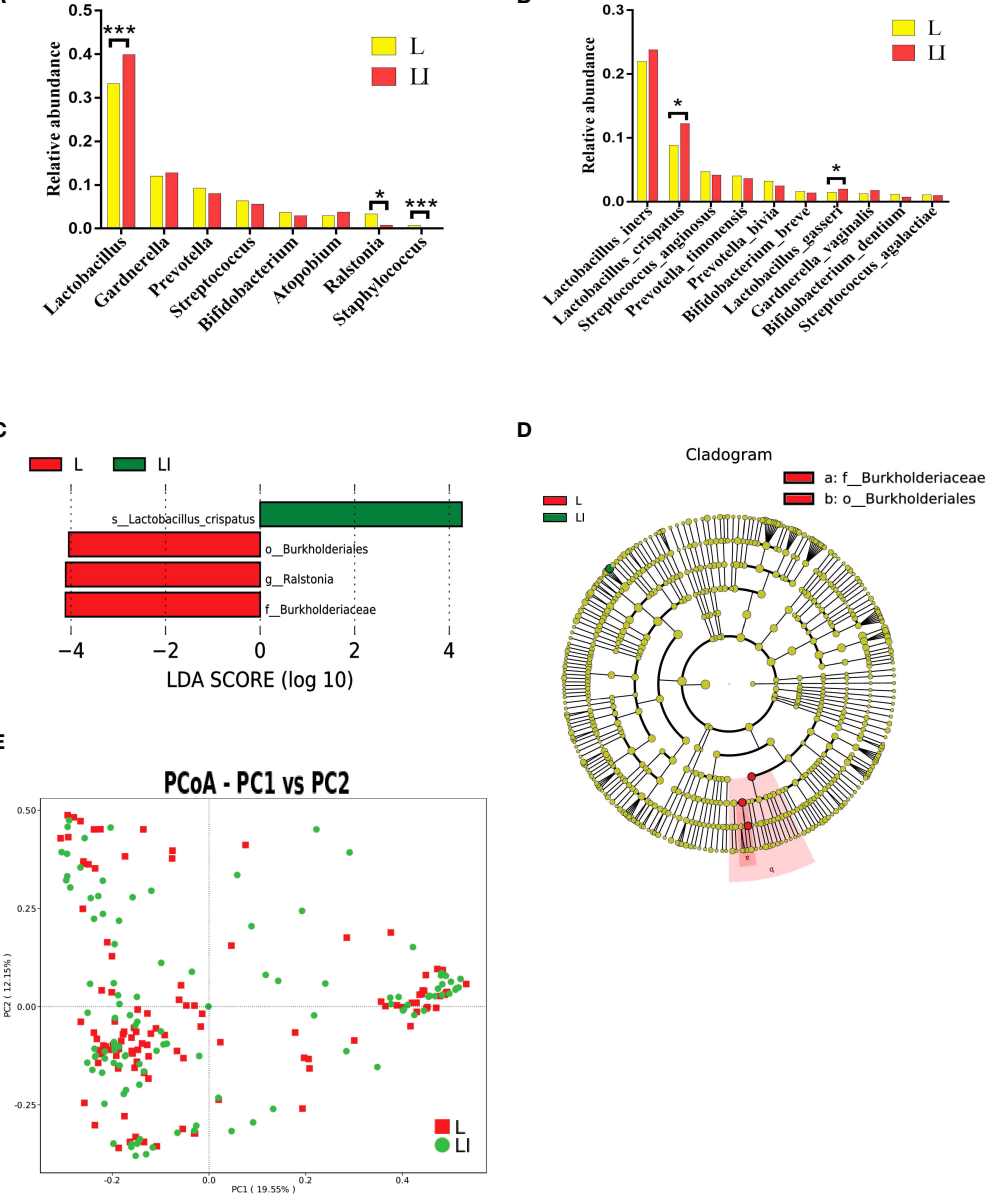
**(A)** Histogram of the major relative abundance of vaginal microbiota at the genus level in L and LI groups. **(B)** Histogram of the major relative abundance of vaginal microbiota at the species level in L and LI groups. *P < 0.05, ***P < 0.001, two-sided paired Wilcoxon rank-sum test. **(C)** LDA scores obtained from the LEfSe of the vaginal microbiota in L and LI groups. An LDA effect size of >4 was used as a threshold for the LEfSe. LDA, linear discriminant analysis; LEfSe, linear discriminant analysis effect size. **(D)** Cladogram of the LEfSe of the vaginal microbiota in L and LI groups. The microbial compositions were compared at different evolutionary levels. **(E)** Principal coordinate analysis of microbial species data based on Bray–Curtis distance matrix which displayed the difference of samples between groups. Each dot represented a sample. L, samples before electrical stimulation in the long protocol cohort; LI, samples after seven times of electrical stimulation in the long protocol cohort.

We defined the characteristics of the 111 participants before ES treatment (L group) as baseline and those after treatment as endpoint (LI group). The relative abundance change of different taxa was used to identify the impacts of ES between L and LI group longitudinally. At the genus level, the relative abundance of *Lactobacillus* spp. was significantly higher in the LI group compared with the baseline (*Z* = -3.540, *P* < 0.001), whereas there was no significant difference of *Gardnerella*, *Atopobium*, *Prevotella* spp., *Bifidobacterium*, and *Streptococcus* between the L and LI groups. Interestingly, *Staphylococcus* and *Ralstonia* spp. that are absent from the healthy vaginal microbiota presented a significant decrease after seven sessions of ES treatments (*Z* = -4.425, *P* < 0.001 and *Z* = -2.016, *P* = 0.044) (**Figure 2A**). We found that *Lactobacillus* was the most common species in our study. Compared with *L. crispatus* (*Z* = -2.519, *P* = 0.012) and *L. gasseri* (*Z* = -2.551, *P* = 0.011) which demonstrated significant increases, that of *L. iners* was somewhat raised but not significantly (**Figure 2B**). The biomarkers between two groups were examined using linear discriminant analysis effect size (LEfSe) to further assess the difference. The Kruskal–Wallis rank sum test (Alpha value = 0.05) was used with the linear discriminant analysis (LDA) score of more than 4, which represented the biomarkers with a statistically significant difference. The *L. crispatus* species were significantly enriched in group LI, whereas the *Burkholderiaceae* family and the *Ralstonia* genus were enriched in group L (**Figures 2C, D**). Next, we utilized MetaStat to analyze other distinct species across groups (**Supplementary Table S4**). Moreover, we demonstrated the differences between L and LI group using PCoA (**Figure 2E**).

Additionally, compared with the baseline, there was a little decrease in the variety and complexity of the vaginal microbiome following ES. The Shannon index was marginally higher in the L group than in the LI group [median 3.32 (IQR 2.45–4.11) *vs*. 3.16 (2.28–3.90)] but not significantly higher (**Supplementary Figure S3A**). The rarefaction curve also revealed the complicacy of the two groups (**Supplementary Figure S3B**).

### Impact of current intensity on vaginal microbiota

To investigate the effect of varied current intensities on vaginal microbiota, we divided the long protocol participants into two groups based on the median electric value of seven sessions of treatments exceeding 40 mA. We discovered that the Shannon index of the low current group displayed a downward trend after ES without significance (*Z* = -1.648, *P* = 0.099), while there was no change in the high current group. At the genus level, we observed that *Lactobacillus* significantly increased (*Z* = -2.227, *P* = 0.026) and *Ralstonia* spp. dramatically decreased (*Z* = -2.908, *P* = 0.004) in the low current group. However, in the high current group, there was a slight increase of *Lactobacillus* without significance (*Z* = -1.592, *P* = 0.111), but there was a significant decrease in *Ralstonia* spp. (*Z* = -2.919, *P* = 0.004) (**Supplementary Figure S2A**). At the species level, only *L. gasseri* (*Z* = -2.652, *P* = 0.008) demonstrated a statistically significant increase in the high current group (**Supplementary Figure S2B**).

### Impact of one session of ES on vaginal microbiota

**Figure 3:**
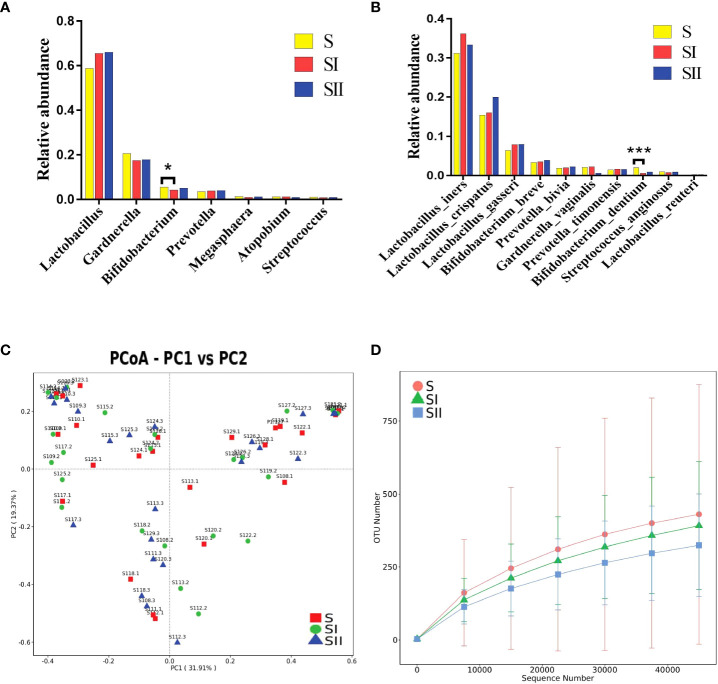
**(A)** Histogram of the major relative abundance of vaginal microbiota at the genus level in S, SI and SII groups. **(B)** Histogram of the major relative abundance of vaginal microbiota at the species level in S, SI and SII groups. *P < 0.05, ***P < 0.001, two-sided paired Wilcoxon rank-sum test. **(C)** Principal coordinate analysis of microbial species data based on Bray–Curtis distance matrix which displayed the difference of samples between groups. All samples were labeled. **(D)** The detected operational taxonomic unit numbers increased with the enlargement of sequence numbers between groups. S, samples before electrical stimulation in the short time cohort; SI, samples after 30 min of electrical stimulation in the short time cohort; SII, samples after electrical stimulation for 7 h in the short time cohort.

To further explore the effects of ES on vaginal microbiota shortly after treatment, 23 women in the short time cohort (S group) were examined. At the genus level, the alterations of the vaginal microbiota following ES were comparable to the long-term cohort but not exactly the same. The relative abundance of *Lactobacillus* spp. in both groups (SI group—30 min after treatment and SII group—7 h after treatment) showed a moderate elevation but without significant difference compared with the S group, whereas *Staphylococcus*, which decreased in the long-term cohort, showed a downward trend, but not significantly, in the short-term cohort (**Figure 3A**). The relative abundance of *Ralstonia* spp. in the short-term cohort was only 0.04%, which was far lower than that in the long-term cohort, and *Ralstonia* spp. did not show a discernible decline. The variations in relative abundance longitudinally at the species level between baseline and after treatment from the same participant were compared, and it demonstrated that *L. crispatus* and *L. gasseri* showed a moderate increase without significance (**Figure 3B**). We also used MetaStat (**Supplementary Table S5**) and PCoA (**Figure 3C**) to further analyze the difference across the three groups.

The alpha diversity variation in the short-term cohort was consistent with that of the long-term cohort, which was slightly lower than the baseline but without significance. We used petal diagram (**Supplementary Figure S3C**) and rarefaction curve to display the differences among the three groups (**Figure 3D**).

### Vaginal cytokines

We ruled out potential secretions containing blood in order to prevent the blood components from interfering with the concentration of local vaginal cytokines. In the end, 117 pairs of cytokine levels were collected, including 94 pairs from the long-term cohort and 23 pairs from the short-term cohort. We examined the expression levels of seven cytokines, including IL-2, IL-4, IL-6, IL-10, TNF-α, IFN-γ, and IL-17A, that are connected to human Th1, Th2, and Th17 immune cells. The percentages of samples within detectable thresholds of cytokine levels were 51.28, 41.45, 95.30, 64.10, 56.84, 53.42, and 52.14%, respectively. The results of the levels of cytokines are presented in **Supplementary Table S6** in great detail.

### Impact of ES on vaginal cytokines

**Figure 4:**
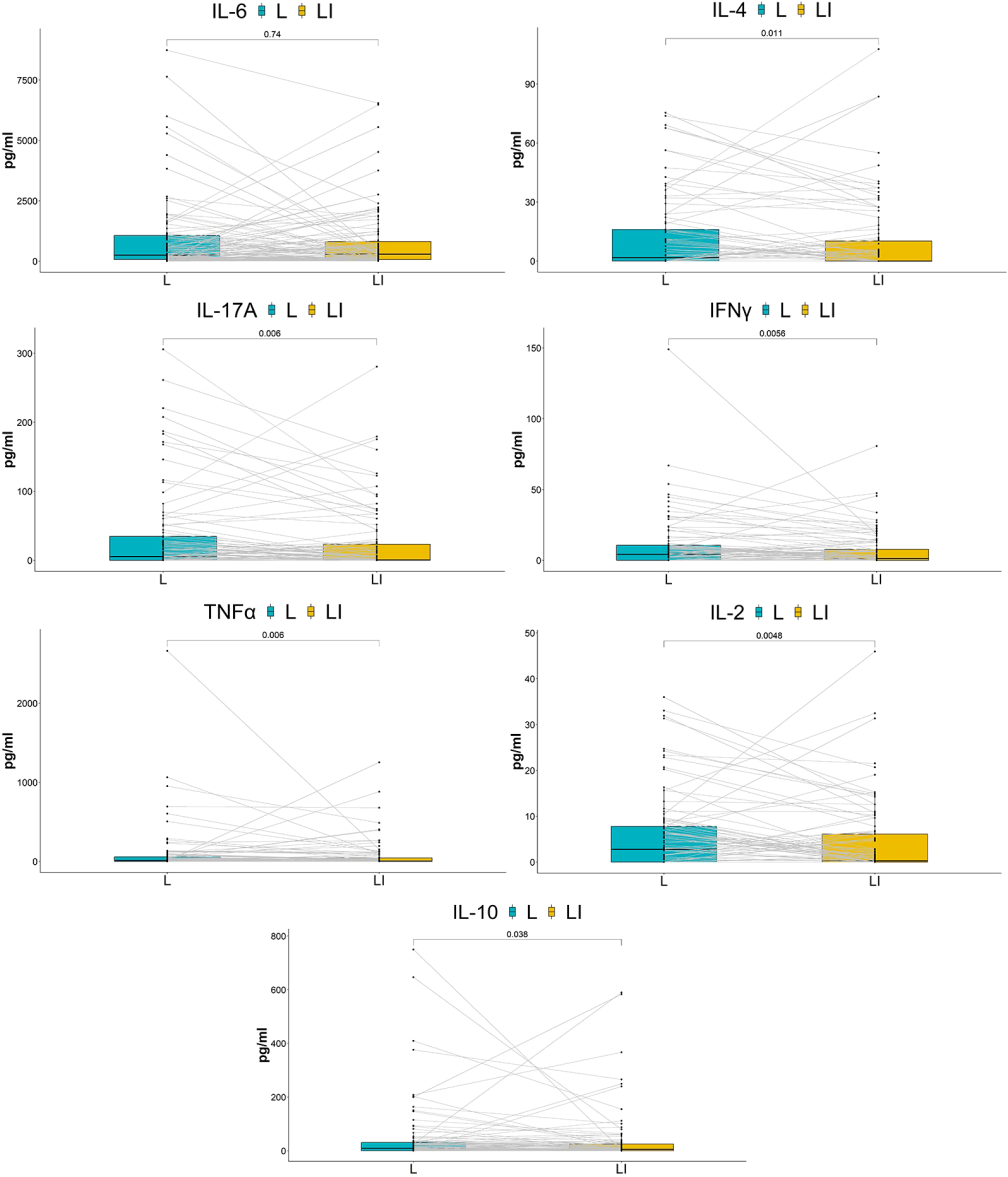
Comparison of cytokine concentrations between baseline and after seven times of treatment. The box plots show the change in cytokine concentrations between groups. *P*-values above the figure were generated using two-sided paired Wilcoxon signed-rank tests. The bounds of boxes show the interquartile range with the lower and upper hinges corresponding to the 25th and 75th percentiles, respectively. Points beyond that were plotted individually.

**Figure 5:**
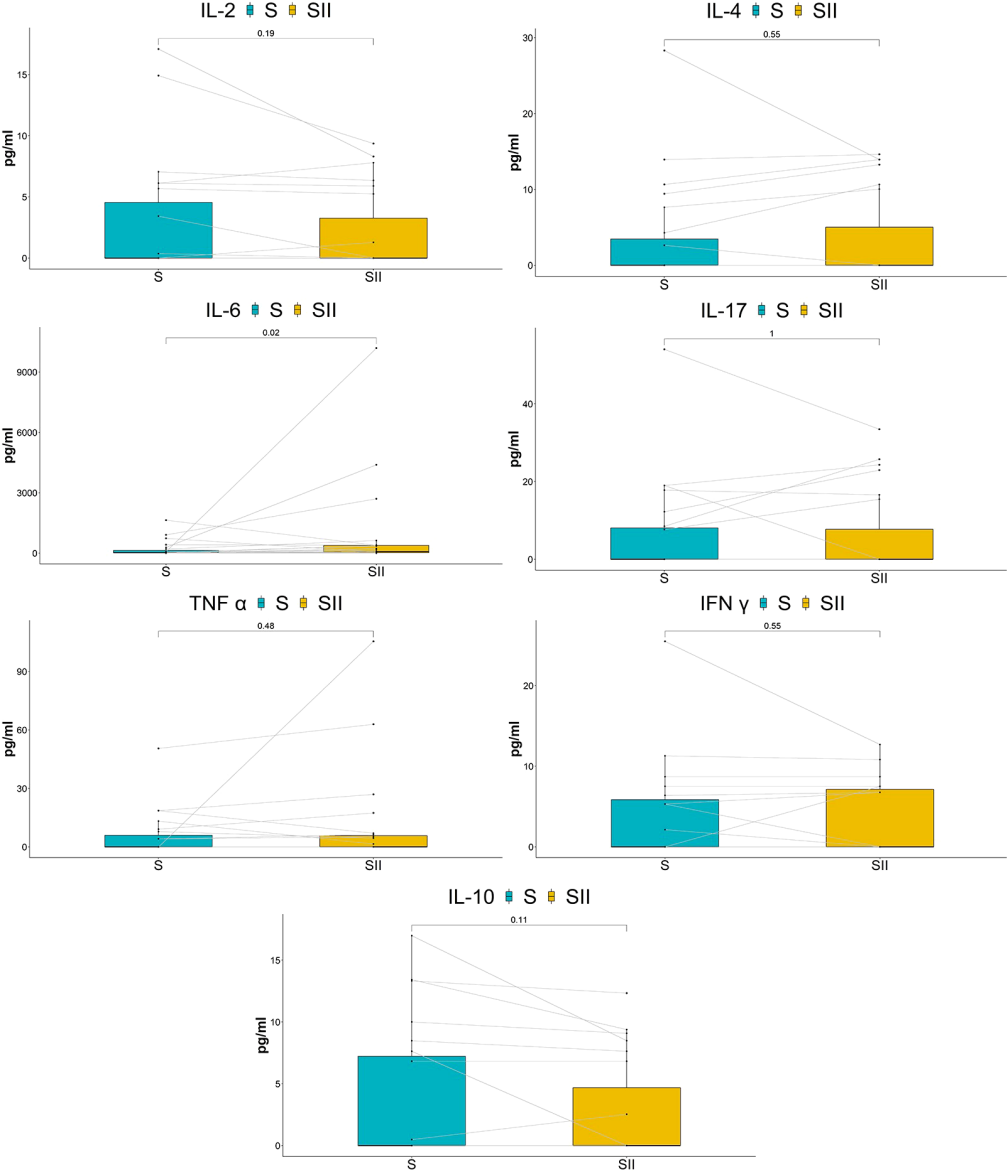
Comparison of cytokine concentrations between baseline and after treatment for 7 h.

Six cytokines (IFN-γ/IL-2/IL-4/IL-17A/TNF-α/IL-10) were significantly downregulated in the long-term cohort compared with the baseline, although IL-6 had no significant change (**Figure 4**). Interestingly, the results of the short-term cohort were opposite to those of the long-term cohort. Compared with baseline, although the IL-2 level showed a trend toward decreased levels, none of the six cytokines (IFN-γ/IL-2/IL-4/IL-17A/TNF-α/IL-10) showed statistically significant changes 7 h after the treatment. On the other hand, IL-6 significantly increased (**Figure 5**).

## Discussion

In recent years, there has been a lot of interest in the studies of pelvic floor ES, vaginal microbiome, and cervico-vaginal immunology. However, there is a lack of research on the relationships between vaginal microbiota, local cytokine levels, and ES. In this longitudinal study, we evaluated both long protocol and short time cohorts to reduce the effects of intercourse and periodic changes of sex hormones on vaginal microbiota and local cytokines (Balle et al., 2020).

In particular, the vaginal microbiota dominated by *L. crispatus*, which produces more lactic acid than *L. iners*, is thought to be more advantageous for keeping the vagina at a low pH level (Pramanick and Aranha, 2020; Adapen et al., 2022; Argentini et al., 2022). *L. crispatus* is related to defense against infections and regulates anti-inflammatory reactions. Microbiomes, dominated by *L. iners*, are more likely to transfer to a diverse microbiome than *L. crispatus* preponderance (Mitra et al., 2020; Breedveld et al., 2022). These mechanisms may account for the rise in *L. crispatus* abundance seen in this study following ES. We observed that the relative abundance of *Lactobacillus* spp., *L. crispatus*, and *L. gasseri* increased after ES. Furthermore, the abundance of *Ralstonia* spp. decreased, and the microbiome diversity slightly declined. These findings suggested that pelvic floor ES might contribute to the preservation of *Lactobacillus* dominance in the vagina, maintaining and possibly supporting vaginal microbiota regeneration to a certain extent. Levy *et al*. discovered that, compared with control rats, rats receiving twice-weekly 30-min sessions of cutaneous ES targeting the genital branch of the pudendal nerve experienced less change in the composition of their vaginal microbiota. This finding suggested that ES might stabilize the vaginal microbiota, which is similar to our conclusion and point (Levy et al., 2020).

While modest inflammatory responses may be beneficial to eradicate various STIs, women with higher vaginal inflammation were more susceptible to HIV exposure, paradoxically increasing the chance of disease acquisition (Gopinath and Iwasaki, 2015). Instantaneously produced in reaction to infection and tissue damage, interleukin-6 (IL-6) contributes to host’s defense against emergent stress by activating acute phase and immune response (Tanaka et al., 2018). In this study, IL-6 significantly increased after a single ES but not after numerous ES, and other cytokines paradoxically decreased. This suggested that pelvic floor ES might not promote local inflammation and may perhaps suppress inflammatory cytokines to some extent. The phenomena deserve to be further studied to explore more applications of ES.

Previous studies showed that the concentrations of pro-inflammatory cytokines were shown to be much lower in women who had cured bacterial vaginosis (BV) than those who were with an infected status, and the levels of cytokines might be influenced by the relative abundance of *G. vaginalis* (Mtshali et al., 2021). Individuals with high-diversity microbiota had significantly higher levels of pro-inflammatory cytokines compared with those with abundant *Lactobacillus*, which is considerable (Anahtar et al., 2015). In our study, six cytokines were downregulated after seven sessions of ES treatments, which may be related to the increase of *Lactobacillus* abundance and the decrease of microbiota diversity. The release of cytokines may also be impacted by the direct influence of ES on immune cells. Further research needs to be done on every possibility. To the best of our knowledge, this study provides the first evidence demonstrating the role of transvaginal ES on microbiota and cytokines, and the first study used 16S rRNA sequencing to explore the effect of direct ES on human organ microbiome. According to these findings, pelvic floor ES might be beneficial to maintain the dominance of *Lactobacillus* in the vagina, reduce the concentration of local cytokines, and even stabilize the vaginal micro-ecology to a certain extent. This study not only fills in the gaps regarding the effect of pelvic floor ES therapy on vaginal microbiome and local inflammation but also suggests a novel approach to the stability of women’s vaginal microenvironment. Additionally, the experimental design was longitudinal—oneself’s comparison at different time points, which reduced interference on results such as individual microbiome difference and time factors, thus improving and enhancing the reliability of the conclusion.

Unfortunately, despite the fact that this study revealed alterations in vaginal flora and local cytokines following ES, there were no descriptions of the production of electrolysis and metabolic substances in the vagina. Changes in chemical substances such as hydrogen peroxide and vaginal pH value should be recorded. Future research may also fill up these gaps to obtain a more thorough understanding of how they interact.

## Methods

### Participants’ enrollment

The study was approved by the Biomedical Ethics Committee of Peking University International Hospital, Beijing, China (ethical approval no. 2021-KY-0017-02). Women undergoing obstetrics and gynecology at Peking University International Hospital as well as healthy volunteers were enrolled in the study. Written consents and face-to-face questionnaires, including relevant information and symptoms, were obtained from all participants. Vaginal secretion samples were collected after the eligibility criteria, instead of exclusion criteria, were confirmed. The eligibility criteria for enrollment in the study included being a married woman with age over 20 years, without intentions to become pregnant throughout the study period, and voluntary signing of the informed consent form and questionnaire. Women who have just given birth are required to have incurred over 6 weeks of rest and without lochia. Additionally, the subjects were required to be free of immune system disorders as well as infectious diseases like hepatitis, AIDS, and syphilis. Furthermore, the participants were instructed to refrain from vaginal irrigation and inserting any drug or objects into the vagina throughout the duration of study and sexual intercourse 48 h before sampling. Participants who use drugs that might influence the vaginal microbiota or inflammatory response during treatment, such as antibiotics, probiotics, and anti-inflammatory medications, were excluded from the study. Moreover, follow-up was ceased in participants who were not having their menstrual period but had interrupted treatments twice or more times.

### ES treatment

In the long protocol cohort, eligible volunteers used TVES for seven sessions for 30 min each, and in the short-term cohort, they only received therapy for 30 min once. There was a 3-day interval between treatments. The volunteers underwent ES treatments for 20 min each, followed by 10 min of Kegel exercise with the assistance of biofeedback. Vishee biostimulation feedback instrument (model SA9804, Nanjing Vishee Medical Technology Co., Ltd., Nanjing, China) and surface EMG electrode (type VET-A, Nanjing Vishee Medical Technology Co., Ltd., Nanjing, China) were used for this study. Supine positions with 45° relaxed abducted hip and knee angles were used for the subjects. Then, an electrode was placed inside the vagina. Each electrode in the long protocol cohort was only utilized by one patient and was rigorously sterilized before and after each treatment. In the short-term cohort, the subjects utilized single-use, sterilized electrodes. The parameters for TVES (constant current generator) were as follows: biphasic pulse, frequency—50  Hz, pulse width—300 μs, on/off—1:1, current intensity: increasing steadily from 0 mA to the highest level tolerable.

### Sample collection

The subjects were placed in lithotomy position on the gynecological examination bed. A well-trained clinical doctor used two disposable sterile swabs to collect secretions by swabbing the vaginal walls three to five times and then removing the swab back into the tube. The samples were maintained in an ice box and transferred to -80°C storage in the laboratory for subsequent experiments.

### DNA extraction

DNA was isolated from vaginal secretions using a Fast DNA Spin Kit (MP Biomedicals, USA). Following the protocol, we added 200 μl of the samples that were submerged in 2 ml saline and 1 ml cell lysis solution (CLS-TC) to Lysing Matrix (FastPrep) and then followed the protocol. Eventually, 50 μl of liquid containing microbial DNA was obtained. DNA concentration and purity were monitored on 1% agarose gel. DNA was diluted in sterile water to 1 g/L depending on the concentration.

### 16S rRNA gene sequencing and analysis

Extracted DNA was amplified by PCR using the 16S V3–V4 primer constructs 806R (5′-GGACTACNNGGGTATCTAAT-3′) and 341F (5′-CCTAYGGGRBGCASCAG-3′). The mixture of PCR products was purified with Qiagen Gel Extraction Kit (Qiagen, Germany). Library preparation was generated using TruSeq^®^ DNA PCR-Free Sample Preparation Kit (Illumina, USA). Finally, the library was sequenced on an Illumina NovaSeq platform, and 250-bp paired-end reads were generated. The paired-end reads were assigned to samples based on their unique barcode and truncated by cutting off the barcode and primer sequence. Paired-end reads were merged using FLASH (V1.2.7, http://ccb.jhu.edu/sofeware/FLASH) (Magoč and Salzberg, 2011). Quality-controlled 16S sequencing reads were processed using the QIIME (V1.9.1, http://qiime.org/scripts/splitlibrariesfastq.html) (Caporaso et al., 2010). The tags were compared with the reference database (Silva database, https://www.arb-silva.de/) using UCHIME algorithm (UCHIME Algorithm, http://www.drive5.com/usearch/manual/uchime_algo.html) to detect and remove chimera sequences (Haas et al., 2011). Then, the effective tags were finally obtained. Sequences analyses were performed by Uparse software (Uparse V7.0.1001, http://drive5.com/uparse/). The sequences were clustered into the same OTUs with 97% similarity or above. For each representative sequence, the Silva Database (http://www.arb-silva.de/) was used based on Mothur algorithm to annotate taxonomic information (Quast et al., 2013). The unannotated sequences were complemented using BLAST on NCBI nucleotide database (excluding uncultured organisms).

### Cytokine measurements

All cytokine levels were measured using bead-based immunoassays (cytometric bead array; CBA) and the CBA Human Th 1/Th 2/Th 17 Cytokine kit (Cat# 560484, BD Biosciences) in accordance with the kit manufacturer’s protocol. Swabs with vaginal secretions were fully mixed after being soaked in tubes with 0.5 ml phosphate buffer saline (HyClone, USA). Cytokine standards were serially diluted 1:2, 1:4, 1:8, 1:16, 1:32, 1:64, 1:128, and 1:256 before mixing with the capture beads and the PE detection reagent. The cytokine standards-bead and samples-bead mixture were incubated for 3 h at room temperature. The beads were analyzed using a BD FACS Calibur flow cytometer (BD Biosciences) after washing. We obtained a standard curve ranging from 0 to 5,000 pg/ml for each cytokine ultimately. The mean fluorescence intensity for each bead cluster was converted into cytokine concentrations based on the 10-point standard curve, and the analysis of data was accomplished by the FCAP Array software (BD version 3.0.1).

### Statistical analysis

Analysis was executed by SPSS version 22 (IBM, New York, NY, USA) and R software (V4.1.2). Continuous data were compared across groups using the Wilcoxon rank-sum test, and statistically significant differences between groups were examined at a two-sided significance level of 0.05. Categorical data were described as frequency proportions. LEfSe software (version 1.0) based on Bray–Curtis was displayed using the Novomagic, a free online platform for data analysis (https://magic.novogene.com). The alpha diversity values were calculated with QIIME software and compared with the Wilcoxon test. Principal coordinate analysis (PCoA) based on Bray–Curtis was manifested by the WGCNA package, stat package, and ggplot2 package in R software. The analysis of cytokines between groups used Wilcoxon rank-sum test by R software.

## Data availability statement

The datasets presented in this study can be found in online repositories. The name of the repository and accession number(s) can be found at: https://www.ncbi.nlm.nih.gov/, PRJNA851474.

## Ethics statement

The studies involving human participants were reviewed and approved by the Biomedical Ethics Committee of Peking University International Hospital, Beijing, China (ethical approval no. 2021-KY-0017-02). The patients/participants provided their written informed consent to participate in this study. Written informed consent was obtained from the individual(s) for the publication of any potentially identifiable images or data included in this article.

## Author contributions

GG and YZ designed the study. YZ and WY performed the acquisition of samples and data. YZ and HY contributed to the laboratory work, data analysis, and plotting of pictures. YZ wrote the original manuscript. GG, CZ, LL, and GX directed and coordinated all aspects of this study. All authors contributed to the review and revision of the manuscript. All authors contributed to the article and approved the submitted version.

## Funding

This work was supported by the University of Chinese Academy of Sciences and Beijing Municipal Science and Technology Commission (grant number: Z181100001818006).

## Acknowledgments

We would like to thank all the participants in this study. We would like to thank the gynecologists and nurses of Peking University International Hospital for their help in this experiment. This trial was supported by the University of Chinese Academy of Sciences through financial provision for this experiment.

## Conflict of interest

The authors declare that the research was conducted in the absence of any commercial or financial relationships that could be construed as a potential conflict of interest.

## Publisher’s note

All claims expressed in this article are solely those of the authors and do not necessarily represent those of their affiliated organizations, or those of the publisher, the editors and the reviewers. Any product that may be evaluated in this article, or claim that may be made by its manufacturer, is not guaranteed or endorsed by the publisher.

## References

[B1] Adapen C. Réot L. Nunez N. Cannou C. Marlin R. Lemaître J.. (2022). Local innate markers and vaginal microbiota composition are influenced by hormonal cycle phases. Front. Immunol. 13, 841723. doi: 10.3389/fimmu.2022.841723 35401577 PMC8990777

[B2] Anahtar M. N. Byrne E. H. Doherty K. E. Bowman B. A. Yamamoto H. S. Soumillon M.. (2015). Cervicovaginal bacteria are a major modulator of host inflammatory responses in the female genital tract. Immunity 42, 965–976. doi: 10.1016/j.immuni.2015.04.019 25992865 PMC4461369

[B3] Argentini C. Fontana F. Alessandri G. Lugli G. A. Mancabelli L. Ossiprandi M. C.. (2022). Evaluation of modulatory activities of lactobacillus crispatus strains in the context of the vaginal microbiota. Microbiol. Spectr. 10, e0273321. doi: 10.1128/spectrum.02733-21 35266820 PMC9045136

[B4] Asadi M. R. Torkaman G. (2014). Bacterial inhibition by electrical stimulation. Adv. Wound Care (New Rochelle) 3, 91–97. doi: 10.1089/wound.2012.0410 24761349 PMC3929208

[B5] Ashrafi M. Baguneid M. Alonso-Rasgado T. Rautemaa-Richardson R. Bayat A. (2017). Cutaneous wound biofilm and the potential for electrical stimulation in management of the microbiome. Future Microbiol. 12, 337–357. doi: 10.2217/fmb-2016-0204 28287302

[B6] Balle C. Konstantinus I. N. Jaumdally S. Z. Havyarimana E. Lennard K. Esra R.. (2020). Hormonal contraception alters vaginal microbiota and cytokines in south African adolescents in a randomized trial. Nat. Commun. 11, 5578. doi: 10.1038/s41467-020-19382-9 33149114 PMC7643181

[B7] Breedveld A. C. Schuster H. J. Van Houdt R. Painter R. C. Mebius R. E. van der Veer C.. (2022). Enhanced IgA coating of bacteria in women with lactobacillus crispatus-dominated vaginal microbiota. Microbiome 10, 15. doi: 10.1186/s40168-021-01198-4 35074009 PMC8787895

[B8] Bruggemann A. K. Mello C. L. Dal Pont T. Hizume Kunzler D. Martins D. F. Bobinski F.. (2017). Effects of neuromuscular electrical stimulation during hemodialysis on peripheral muscle strength and exercise capacity: A randomized clinical trial. Arch. Phys. Med. Rehabil. 98, 822–831.e1. doi: 10.1016/j.apmr.2016.12.009 28093194

[B9] Caporaso J. G. Kuczynski J. Stombaugh J. Bittinger K. Bushman F. D. Costello E. K.. (2010). QIIME allows analysis of high-throughput community sequencing data. Nat. Methods 7, 335–336. doi: 10.1038/nmeth.f.303 20383131 PMC3156573

[B10] Caubet R. Pedarros-Caubet F. Chu M. Freye E. De Belém Rodrigues M. Moreau J. M.. (2004). A radio frequency electric current enhances antibiotic efficacy against bacterial biofilms. Antimicrob. Agents Chemother. 48, 4662–4664. doi: 10.1128/AAC.48.12.4662-4664.2004 15561841 PMC529182

[B11] Chee W. J. Y. Chew S. Y. Than L. T. L. (2020). Vaginal microbiota and the potential of lactobacillus derivatives in maintaining vaginal health. Microb. Cell Fact 19, 203. doi: 10.1186/s12934-020-01464-4 33160356 PMC7648308

[B12] Chen Y. Qiu X. Wang W. Li D. Wu A. Hong Z.. (2020). Human papillomavirus infection and cervical intraepithelial neoplasia progression are associated with increased vaginal microbiome diversity in a Chinese cohort. BMC Infect. Dis. 20, 629. doi: 10.1186/s12879-020-05324-9 32842982 PMC7449047

[B13] Dusane D. H. Lochab V. Jones T. Peters C. W. Sindeldecker D. Das A.. (2019). Electroceutical treatment of pseudomonas aeruginosa biofilms. Sci. Rep. 9, 2008. doi: 10.1038/s41598-018-37891-y 30765750 PMC6375951

[B14] Gentzkow G. D. (1993). Electrical stimulation to heal dermal wounds. J. Dermatol. Surg. Oncol. 19, 753–758. doi: 10.1111/j.1524-4725.1993.tb00420.x 8349916

[B15] Gopinath S. Iwasaki A. (2015). Cervicovaginal microbiota: simple is better. Immunity 42, 790–791. doi: 10.1016/j.immuni.2015.05.006 25992855

[B16] Haas B. J. Gevers D. Earl A. M. Feldgarden M. Ward D. V. Giannoukos G.. (2011). Chimeric 16S rRNA sequence formation and detection in Sanger and 454-pyrosequenced PCR amplicons. Genome Res. 21, 494–504. doi: 10.1101/gr.112730.110 21212162 PMC3044863

[B17] Kopitar A. N. Kotnik V. Vidmar G. Ihan A. Novak P. Stefancic M. (2012). Therapeutic electric stimulation does not affect immune status in healthy individuals - a preliminary report. BioMed. Eng. Online 11, 42. doi: 10.1186/1475-925X-11-42 22839574 PMC3444347

[B18] Lee M. H. Jeong H. Koo M. A. Seon G. M. Hong S. H. Park Y. J.. (2021). Sterilization of sealed PVDF pouches containing decellularized scaffold by electrical stimulation. Biotechnol. J. 16, e2100156. doi: 10.1002/biot.202100156 34374222

[B19] Levy M. Bassis C. M. Kennedy E. Yoest K. E. Becker J. B. bell J.. (2020). The rodent vaginal microbiome across the estrous cycle and the effect of genital nerve electrical stimulation. PloS One 15, e0230170. doi: 10.1371/journal.pone.0230170 32163469 PMC7067422

[B20] Li W. Hu Q. Zhang Z. Shen F. Xie Z. (2020). Effect of different electrical stimulation protocols for pelvic floor rehabilitation of postpartum women with extremely weak muscle strength: Randomized control trial. Med. (Baltimore) 99, e19863. doi: 10.1097/MD.0000000000019863 PMC7440138 32332648

[B21] Magoč T. Salzberg S. L. (2011). FLASH: fast length adjustment of short reads to improve genome assemblies. Bioinformatics 27, 2957–2963. doi: 10.1093/bioinformatics/btr507 21903629 PMC3198573

[B22] Mitchell C. Marrazzo J. (2014). Bacterial vaginosis and the cervicovaginal immune response. Am. J. Reprod. Immunol. 71, 555–563. doi: 10.1111/aji.12264 24832618 PMC4128638

[B23] Mitra A. Macintyre D. A. Ntritsos G. Smith A. Tsilidis K. K. Marchesi J. R.. (2020). The vaginal microbiota associates with the regression of untreated cervical intraepithelial neoplasia 2 lesions. Nat. Commun. 11, 1999. doi: 10.1038/s41467-020-15856-y 32332850 PMC7181700

[B24] Mtshali A. San J. E. Osman F. Garrett N. Balle C. Giandhari J.. (2021). Temporal changes in vaginal microbiota and genital tract cytokines among south African women treated for bacterial vaginosis. Front. Immunol. 12, 730986. doi: 10.3389/fimmu.2021.730986 34594336 PMC8477043

[B25] Otani S. Fujii T. Kukimoto I. Yamamoto N. Tsukamoto T. Ichikawa R.. (2019). Cytokine expression profiles in cervical mucus from patients with cervical cancer and its precursor lesions. Cytokine 120, 210–219. doi: 10.1016/j.cyto.2019.05.011 31121496

[B26] Pramanick R. Aranha C. (2020). Distinct functional traits of lactobacilli from women with asymptomatic bacterial vaginosis and normal microbiota. Microorganisms 8 , 12949.9. doi: 10.3390/microorganisms8121949 PMC7763271 33316918

[B27] Quast C. Pruesse E. Yilmaz P. Gerken J. Schweer T. Yarza P.. (2013). The SILVA ribosomal RNA gene database project: improved data processing and web-based tools. Nucleic Acids Res. 41, D590–D596. doi: 10.1093/nar/gks1219 23193283 PMC3531112

[B28] Ravel J. Gajer P. Abdo Z. Schneider G. M. Koenig S. S. Mcculle S. L.. (2011). Vaginal microbiome of reproductive-age women. Proc. Natl. Acad. Sci. U.S.A. 108 (Suppl 1), 4680–4687. doi: 10.1073/pnas.1002611107 20534435 PMC3063603

[B29] Silantyeva E. Zarkovic D. Astafeva E. Soldatskaia R. Orazov M. Belkovskaya M.. (2021). A comparative study on the effects of high-intensity focused electromagnetic technology and electrostimulation for the treatment of pelvic floor muscles and urinary incontinence in parous women: Analysis of posttreatment data. Female Pelvic Med. Reconstr Surg. 27, 269–273. doi: 10.1097/SPV.0000000000000807 31860567 PMC8016513

[B30] Tanaka T. Narazaki M. Kishimoto T. (2018). Interleukin (IL-6) immunotherapy. Cold Spring Harb. Perspect. Biol. 10 , 8 a028456. doi: 10.1101/cshperspect.a028456 PMC6071487 28778870

[B31] Wang T. Q. Li L. R. Tan C. X. Yang J. W. Shi G. X. Wang L. Q.. (2021). Effect of electroacupuncture on gut microbiota in participants with knee osteoarthritis. Front. Cell Infect. Microbiol. 11, 597431. doi: 10.3389/fcimb.2021.597431 34671567 PMC8521167

[B32] Yang S. Sang W. Feng J. Zhao H. Li X. Li P.. (2017). The effect of rehabilitation exercises combined with direct vagina low voltage low frequency electric stimulation on pelvic nerve electrophysiology and tissue function in primiparous women: A randomised controlled trial. J. Clin. Nurs. 26, 4537–4547. doi: 10.1111/jocn.13790 28252827

