# Supplementary figures and images for: The impact of pelvic floor electrical stimulation on vaginal microbiota and immunity
Source: Front Cell Infect Microbiol. 2022 Sep 27;12:1006576. doi: 10.3389/fcimb.2022.1006576 (PMC9551273; doi:10.3389/fcimb.2022.1006576)

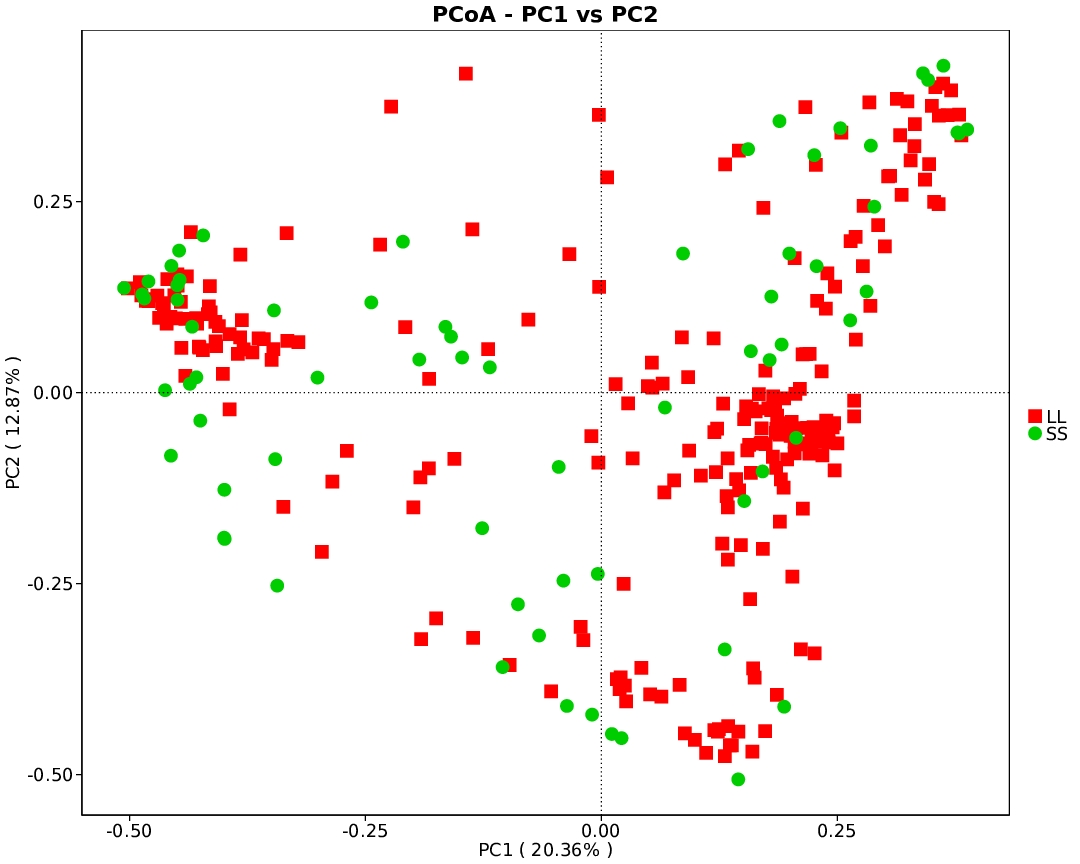

Supplement: Supplementary file 1 [file Image_1.jpeg]

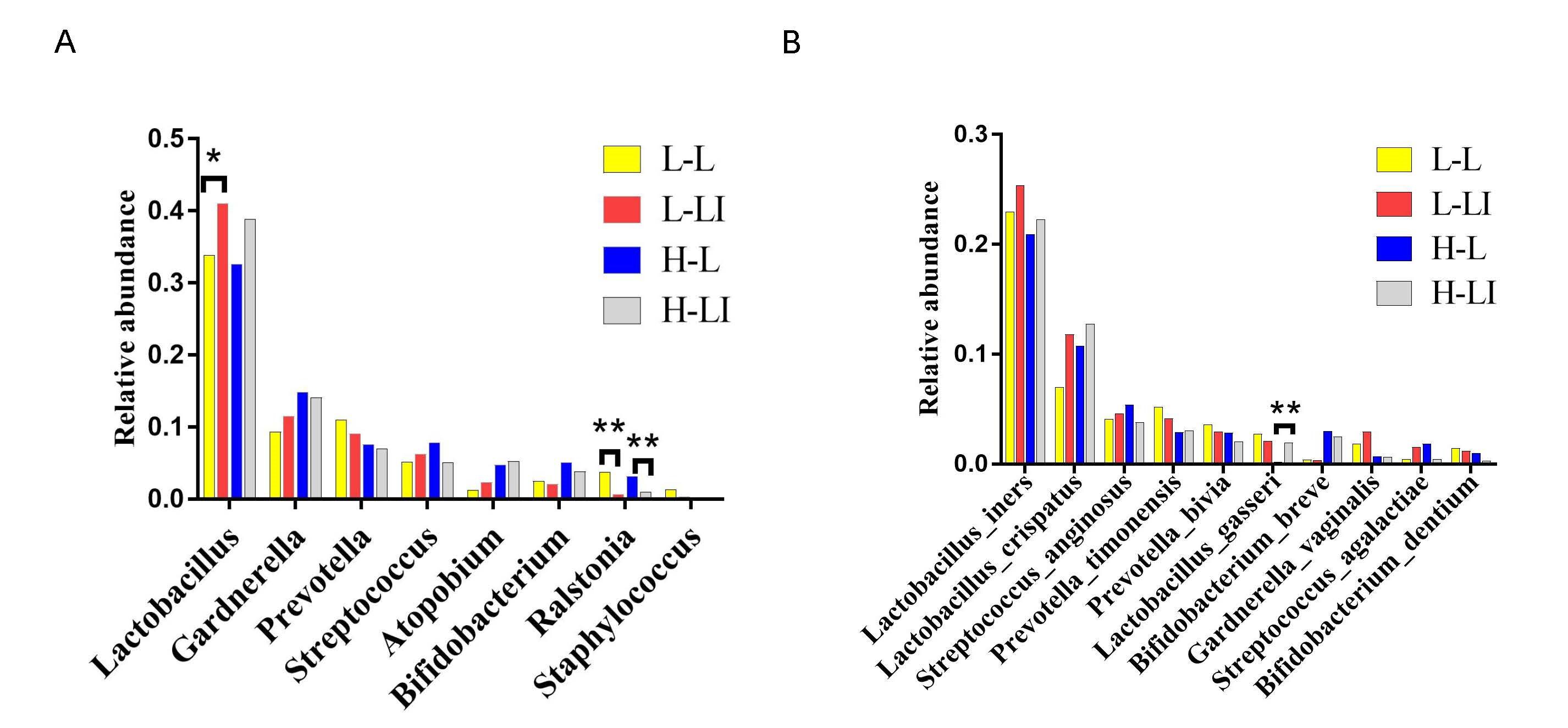

Supplement: Supplementary file 2 [file Image_2.jpg]

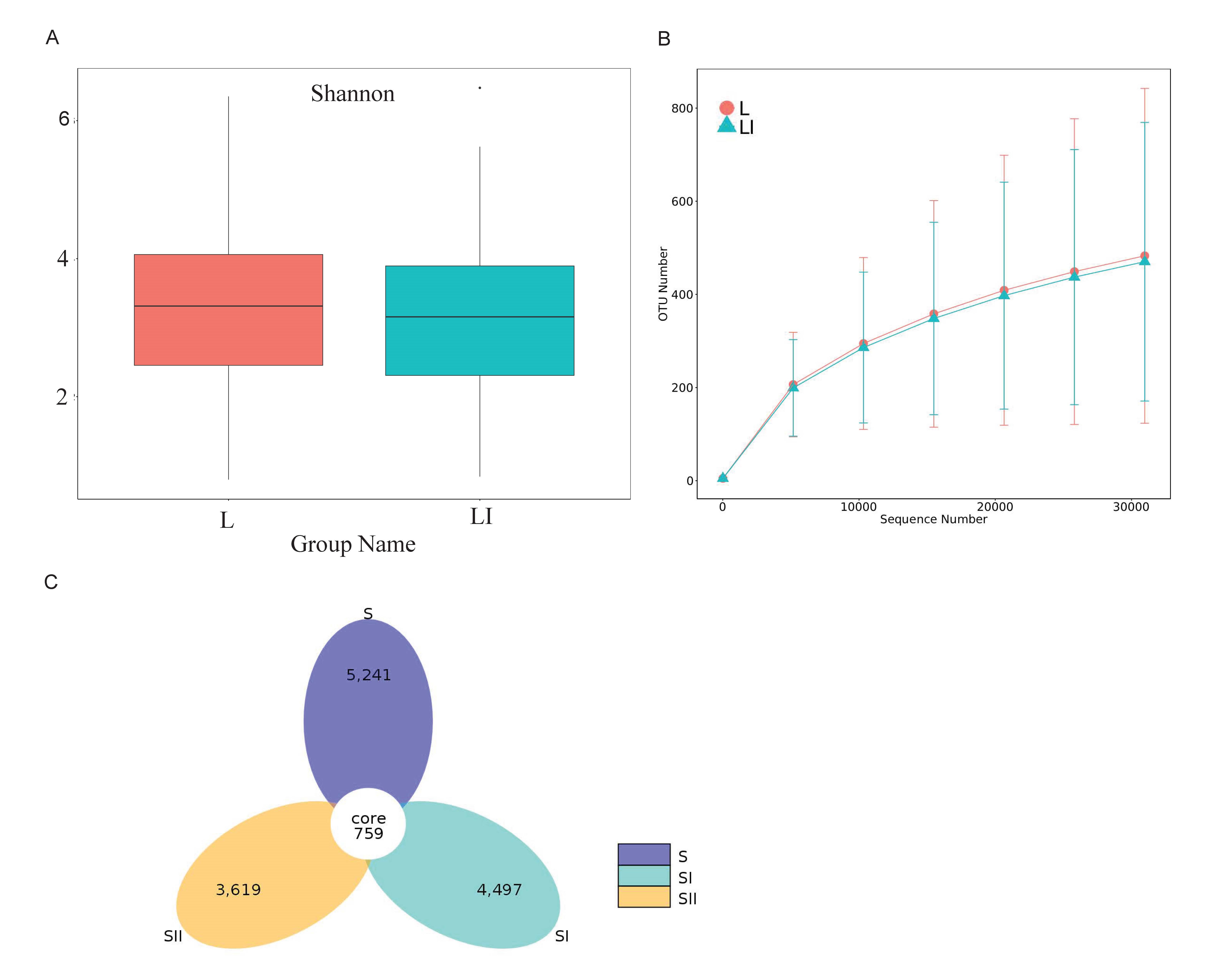

Supplement: Supplementary file 3 [file Image_3.jpeg]
